# An exploratory study into the impact and acceptability of formatively used progress testing in postgraduate obstetrics and gynaecology

**DOI:** 10.1007/s40037-013-0063-2

**Published:** 2013-06-21

**Authors:** Marja G. K. Dijksterhuis, Lambert W. T. Schuwirth, Didi D. M. Braat, Fedde Scheele

**Affiliations:** 1 Department of Obstetrics and Gynaecology, Amphia Hospital, Langendijk 75, 4918 EV Breda, the Netherlands; 2 Flinders Innovation in Clinical Education, Health Professions Education, School of Medicine Flinders University, Adelaide, SA Australia; 3 Department of Obstetrics and Gynaecology, University Medical Center Nijmegen, Nijmegen, the Netherlands; 4 Institute for Medical Education and Training, VU University Medical Centre, Amsterdam, the Netherlands

**Keywords:** Postgraduate, Formative assessment, Progress test, Acceptability, Educational impact

## Abstract

Part of recent reforms of postgraduate medical training in the Netherlands is the introduction of formatively intended knowledge testing or progress testing. We previously evaluated the construct validity and reliability of postgraduate progress testing. However, when assessment is intended to be formative, the acceptability of the test (scores) and the educational impact that is achieved are at least as important in the utility of this assessment format. We developed a questionnaire targeted at both educational supervisors and postgraduate trainees, containing questions on general acceptability, educational impact and acceptability of test content. 90 % of trainees and 84 % of educational supervisors completed the questionnaire. The general acceptability of formatively used progress testing is good; however, the self-reported educational impact is limited. Furthermore, trainees query the validity of test content. Formatively intended progress testing is well accepted; however the impact is limited. We discuss the importance of feedback quality and the effect of grading. Furthermore we start a debate on whether, for a genuine effect on learning, formative assessment should have consequences, either by entwining the assessment with the training programme or by linking the assessment to a summative standard.

## Introduction

Like many other postgraduate training programmes worldwide, postgraduate training in the Netherlands has recently been the subject of major reforms by the Dutch Legislative Board of Medical Specialists (CGS). Part of these reforms has been a complete restructuring of the postgraduate assessment system: from ‘just’ loosely structured yearly in-training assessments with the educational supervisor to a comprehensive assessment system adding knowledge testing, workplace-based assessments and a portfolio [1].

For knowledge testing, the CGS selected the progress test format. This test format was initially developed to prevent test-driven learning behaviour in problem-based undergraduate curricula [2, 3]. For this reason, the test is attuned to the postgraduate level and encompasses all subjects that are to be covered during the postgraduate curriculum. This renders the test too comprehensive for last-minute test preparation; however, all pertinent study efforts should be rewarded, preparing the student for a career of lifelong learning.

Usually, progress tests are taken at regular intervals during the year by all students of all year classes, enabling a good impression of the knowledge growth of students. Furthermore, test results provide a rich source of feedback which can help both students and teachers in identifying individual learning goals. Both factors—test-driven study behaviour not being encouraged and progress testing having a rich formative potential (providing feedback that can assist learning)—were reasons for the CGS to select the progress test format for postgraduate knowledge testing. Additionally, the CGS decreed that solely failing the progress test should not have consequences for the progression of trainees as knowledge represents only part of the clinical competence of trainees [1].

In postgraduate obstetrics and gynaecology (O&G) progress testing was introduced back in 1999 and, so far, this training programme has the most experience with postgraduate progress testing in the Netherlands. We analyzed the reliability and validity of postgraduate progress testing in an earlier paper, finding a reasonable reliability (Cronbach’s alpha 0.65) and low construct validity (no significant knowledge growth from training year 2 onwards) [4]. Efforts to improve these test characteristics, by changing from true–false questions to multiple choice questions and more emphasis on knowledge questions that are relevant to daily clinical practice, only somewhat improved reliability, without having an effect on construct validity. Furthermore, we found a strikingly similar answer pattern year after year, where the number of correctly answered questions increases over training years and the number of question marks decreases, but with a stable percentage (25–30 %) of incorrectly answered questions for every training year and every test.

However, as the goal of formatively used assessment is to stimulate learning, psychometrics are not the only determinants of utility. It can be argued that acceptability to stakeholders and the educational impact that is achieved are at least as important [5–7]. In this perspective, acceptability can be defined as the degree to which stakeholders support the test and the associated interpretation of scores [8]. Educational impact, on the other hand, is the extent to which the feedback resulting from assessment becomes an integral part of the learning process and influences trainee learning behaviour. Even though formative assessment has taken a flight during postgraduate training internationally [9–11], evidence on the acceptability and/or educational impact of this test format is still hard to find [12, 13].

We wondered whether the disappointing psychometrics of our progress test could coincide with the acceptability and/or educational impact of our test. For this reason we set out to complement our earlier study by performing a preliminary study into acceptability and educational impact of formatively intended knowledge testing. Both postgraduate trainees and their educational supervisors were approached for this study as we postulated that triangulation would generate a more complete picture of the acceptability and educational impact of the progress test and that divergences in answer patterns could be helpful in improving the utility of the progress test.

## Methods

### Educational context

In the Netherlands, postgraduate training in O&G is a 6-year programme consisting of clinical rotations complemented by formal teaching. Trainees can start at any moment throughout the year, as the Netherlands does not have an ‘academic year’ in postgraduate medical training. There are no formal licensing exams at the end of training; the progress of trainees is monitored and assessed through the trainee’s portfolio. The portfolio contains the results of formative workplace-based assessments and the progress knowledge test, numbers of performed procedures and reports of the in-training assessments with the educational supervisor. Taking the yearly progress test is a mandatory part of postgraduate training, though substandard performance does not have formal consequences [1].

### Dutch postgraduate progress test for O&G

Each individual progress test consists of approximately 150 multiple-choice questions. Questions are divided over five O&G sub-domains according to a predetermined blueprint (perinatology, benign gynaecology, reproduction and endocrinology, oncology and general health). Every year the progress test exam committee constructs a new set of exam questions based on recently released practice guidelines, recent relevant research findings and knowledge that is relevant for daily clinical practice. The exam committee consists of 8–10 practising gynaecologists who all represent one or more sub-domains. Furthermore, each year two different trainees are invited to participate in test construction. They have the final say in whether test questions are relevant enough to daily clinical practice to be included in the test. Following the test all participating trainees are given the opportunity to leave comments on test questions or answer keys, and when their objections are valid, test questions will be omitted.

Trainees receive 1 point for a correct answer, and 1 point is subtracted for an incorrect answer. Trainees can also choose a question mark, in which case 0 points are given. Total test scores and results on the five sub-domains are fed back to both educational supervisors and trainees. Additionally, a norm-referenced pass/fail judgment is provided, and a score less than 2 standard deviations below the year group mean (≈<2.3rd percentile) is considered ‘unsatisfactory’. But, as the test is intended to be formative, unsatisfactory test results do not have formal consequences. After the test, trainees are allowed to take booklets containing the questions of the test home. To optimize the formative function of the test and stimulate learning, trainees do not receive answer keys but are instead challenged to look for the correct answers in the literature and debate their findings with peers and supervisors.

Graduated gynaecologists are invited to voluntarily take the progress test as part of their personal continuing medical education programme. Following the test all test questions are placed on the members-only section of the Dutch Society of Obstetricians and Gynaecologists (NVOG) website to give non-participating members insight into the test content and provide them with an opportunity to test their knowledge.

For a more detailed description of the progress test in postgraduate O&G training we refer to our earlier paper [4].

### Questionnaire acceptability and impact

An extensive search in PubMed and Google scholar using the search terms: “acceptability”, “impact”, “assessment” and “(post) postgraduate education” did not provide any questionnaires suitable for measuring acceptability and educational impact of an educational intervention in postgraduate medical training. Therefore we developed two questionnaires, one for trainees and one for educational supervisors.

Draft versions of the questionnaire were presented to six experts. Four were gynaecologists with extensive experience with progress testing, one was a medical educationalist and one was a psychometrician. Their comments were used to adapt the draft into the final version of the questionnaire.

The trainee questionnaire consisted of eight questions on acceptability, covering both general acceptability (4) and acceptability of test content (4) and ten questions on educational impact. Seventeen questions consisted of five-point Likert scale statements, in which 1 represented ‘disagree’ and 5 represented ‘agree’, 3 is neutral, neither agree nor disagree; one item was a multiple-choice question. At the end of the questionnaire one free-response item was included asking how progress testing could be improved.

The questionnaire for educational supervisors comprised four questions on general acceptability (we assumed that the majority of supervisors would not have recent information on test content) and nine questions on educational impact. The questions of this questionnaire were the same as the questions for trainees. Twelve questions could be answered by a similar Likert scale: one item was a multiple-choice question. At the end of the questionnaire a free-response item was included, asking how progress testing could be improved.

### Participants

All postgraduate trainees (263) taking the yearly formative knowledge test of April 2010 were asked to anonymously complete the questionnaire at the end of the test, before they were aware of their results. A total of 236 (90 %) completed the questionnaire. At the same time all educational supervisors (45) received an anonymous postal questionnaire. A follow-up reminder was sent 3 weeks later. Thirty-eight (84 %) returned a completed questionnaire.

### Test scores 2010

The final test consisted of 151 questions in 2010. Table 1 displays test scores per training year for all trainees who took the progress test in 2010. As we guaranteed anonymity, we are not able to correlate individual test scores to the answers on the educational impact an acceptability questionnaire.

**Table 1:** Mean test results (% correct–incorrect) per training year and number of failures per training year

Training year	*N*	Min	Max	Mean	SD	Fail
1	49	15	52	32	8.5	0
2	46	23	60	41	8.1	1
3	41	29	66	45	9.5	0
4	51	−14	63	46	12	1
5	24	27	59	45	7.4	1
6	51	28	67	47	8.7	0

Table 2 shows the answer patterns for correct, incorrect and question mark answers. Yearly analysis of former progress tests shows a similar pattern: the number of correctly answered questions increases, the number of question marks decreases, but the number of incorrect answers remains stable at 25–30 %.

**Table 2:** Mean number of correct, incorrect and question mark answers per training year

Training year	Correct mean number	Incorrect mean number	Question mark mean number
1	64	46	41^a^
2	78	45	28
3	83	42	25
4	85	45	20
5	85	45	21
6	89	50	12

^a^The questionnaire consisted of 151 questions in 2010

### Statistical analysis

Although a Likert scale is an ordinal scale, following common practice descriptive statistics are presented as means and standard deviations for the whole group of trainees and educational supervisors. However, this method of data handling may mask meaningful differences in answer patterns, especially between different training years of the trainees. For this reason we also provided mean scores per training year. Furthermore we tested for which questions trainees’ and educational supervisors’ results differed significantly by performing a Mann–Whitney test (as group sizes were not equal and data were skewed). To test for differences in answer patterns between year groups we performed a one-way ANOVA with post hoc Scheffé’s test.

The open-ended questions, which asked for suggestions on how to improve postgraduate progress testing, were completed by 87 trainees (35 %) and ten (26 %) educational supervisors. Responses could be grouped in suggestions on how to improve test content and on how to improve educational impact.

## Results

Mean scores for the complete group of trainees and mean scores per training year, standard deviations and significant differences between trainees and educational supervisors are presented in Table 3. Except for the question on fairness of the pass/fail standard, all questions in Table 3 used a Likert scale, 1 representing disagree, 5 representing agree. Mean scores should be interpreted similarly: the closer to 1, the more the group disagreed, the closer the mean score to 5, the more the group agreed with the statement. As not all trainees/educational supervisors answered all questions, the number of respondents varies per question.

**Table 3:** Results of trainees’ and educational supervisors’ questionnaire on acceptability and educational impact

	Supervisor: mean (*n*, SD)	Trainees: mean (*n*, SD)	Significance Mann–Whitney test (*p*)	Training year						Significance One-way Anova + post hoc test
				1	2	3	4	5	6	
*General acceptance*										
The progress test should continue to be obligatory	4.5 (38, 0.9)	3.4 (205, 1.2)	0.000	3.8	3.5	3.3	3.0	3.2	3.2	0.118
I believe that our society deserves an adequate exam policy during medical specialist training	4.2 (38, 0.9)	3.7 (206, 1.0)	0.000	3.6	3.7	4.0	3.5	3.8	3.5	0.393
The pass–fail standard is: 1. to low, 2. fair, 3. to high	1.8 (32, 0.4)	2.1 (158, 0.4)	0.000	2.1	2.2	2.1	2.2	2.0	1.9	0.088
This kind of testing within adult education is belittling	1.7 (38, 0.8)	2.4 (236, 1.1)	0.000	1.8	2.4	2.5	2.6	2.3	2.6	0.004 (6 + 4-rest)
*Educational impact*										
The progress test is a major assessment of my development/the development of the trainee	3.6 (38, 0.7)	2.7 (214, 1.0)	0.000	3.1	2.9	2.5	2.6	2.3	2.4	0.001 (1–2)
The progress test could be helpful in compiling my learning course/the learning course of the trainee together with my supervisor	4.0 (38, 0.9)	3.0 (236, 1.1)	0.000	3.6	3.2	2.7	2.8	2.8	3.0	0.01 (1–2)
The results of the progress test should be discussed during the in-training assessment with the educational supervisor	4.7 (38, 0.5)	3.6 (234, 0.9)	0.000	3.8	3.6	3.4	3.6	3.5	3.4	0.519
The progress test helps me to get a good impression of the functional knowledge of the trainee	3.5 (38, 0.9)	3.2 (206, 1.0)	n.s.	3.8	3.5	2.9	3.0	2.9	2.9	0.000 (1–2)
The results of the progress test increase my self-confidence		2.7 (228, 1.0)	n.a.	2.6	2.7	2.8	2.6	2.8	2.8	0.864
I use the progress test to compile my learning course together with my educational supervisor	3.4 (38, 1.1)	1.9 (235, 0.9)	0.000	2.5	1.9	1.7	2.0	1.6	1.8	0.000 (1 + 4-rest)
The progress test is a predominant part of my in-training assessment	2.5 (38, 0.9)	1.8 (205, 0.8)	0.000	2.2	1.7	1.7	2.0	1.6	1.5	0.001 (1 + 4-rest)
The results on the different test domains influence my study activities		3.0 (205, 1.1)	n.s.	3.7	3.1	2.7	3.0	2.7	2.6	0.000 (1 + 4-rest)
The results of the progress test influence my training programme	3.0 (38, 1.0)		n.s.							
I/trainees need this kind of stimulus in order to study	4.2 (38, 0.8)	2.7 (235, 1.3)	0.000	2.7	3.0	2.5	2.8	2.8	2.7	0.496
The test results should have more serious consequences for the training progression of a trainee	2.9 (38, 1.1)	2.0 (206, 1.0)	0.000	1.9	1.9	1.9	2.0	1.8	2.2	0.719

	Supervisor: mean (*n*, SD)	Trainees: mean (*n*, SD)	Training year						Significance One-way Anova + post hoc test
			1	2	3	4	5	6	
*Acceptability of test content*									
The progress test properly reflects the whole domain of O&G	–	3.4 (234, 1.0)	3.6	3.3	3.2	3.2	3.4	3.4	0.563
The distribution of the questions over the sub-domains of O&G reflects daily practice	–	3.5 (204, 1.0)	3.3	3.2	3.6	3.4	3.2	3.2	0.580
The progress test is a good instrument to test knowledge	–	2.9 (205, 1.1)	3.5	3.2	2.7	2.8	2.8	2.6	0.000 (1–2)
The progress test tests the knowledge that is needed to be able to work as a gynaecologist	–	2.4 (236, 1.0)	3.0	2.5	2.2	2.3	2.3	2.3	0.002 (1–2)

*n.s.* not significant

The one-way ANOVA with post hoc Scheffé’s test mainly shows differences in answer patterns between first-year trainees and older trainees: younger trainees appear to have a more positive outlook on progress testing than their older counterparts. For example, they agree more often with the statements: ‘the progress test could be helpful in compiling my learning course together with my supervisor (mean 3.6–2.9)’; ‘the results on the different sub-domains influence my study activities (mean 3.0–2.5)’ and ‘the progress test is a good instrument to test knowledge (mean 3.5–2.8)’.

### General acceptability

Educational supervisors (es) rate the general acceptability of progress testing higher than trainees (t), though both groups agree that progress testing should remain mandatory (4.5 (es); 3.4 (t)) and that society is entitled to medical speciality training programmes with an adequate exam policy (4.2 (es); 3.7 (t)). Also, both groups deem the pass-fail standard to be fair (1.8 (es); 2.1 (t)) (1 = too low, 2 = fair, 3 = too high) and both groups largely disagree with the statement that this kind of testing within adult education is belittling (1.7 (es); 2.4 (t)).

### Educational impact

Educational supervisors tend to place more weight on progress test results than trainees: they agree more often with the statement that progress testing is a major assessment of the development of a trainee (mean scores 3.6 (es); 2.7 (t)), that the progress test could be helpful in compiling the learning course of the trainee (4.0 (es); 3.0 (t)) and that progress test results should be discussed during the in-training assessment (4.7 (es); 3.6 (t)). Both agree somewhat with the statement that the progress test helps to get a good impression of the functional knowledge of the trainee (3.5 (es); 3.2 (t)). However, trainees do not report an effect on their self-confidence (2.7). Additionally, regarding the discussion of test results during the in-training assessment both educational supervisors and trainees report a discrepancy between their intentions and actual practice: progress test results are not always used to compile the learning course of the trainee (3.4 (es); 1.9 (t)) and certainly do not play a predominant role during in-training assessments (2.5 (es); 1.8 (t). Both react equivocally to the statement about whether test results influence the training programme or study activities (3.0 (es); 3.0 (t). Finally educational supervisors strongly agree with the statement that trainees need the stimulus from exams to start studying (4.2 (es); 2.7 (t)), even though neither they nor the trainees would like test results to have more serious consequences for the progress of the trainee (2.9 (es); 2.0 (t).

### Acceptability of test content

When trainees were asked about the validity of the test, or whether they have the impression that the test really measures what it is supposed to measure, they appear to be moderately satisfied with the test content (means per question ranging from 2.4 to 3.4). They agree least with the statement that the progress test tests the knowledge that is needed to be able to work as a gynaecologist (mean 2.4).

### Open questions

The answers that were given in response to the open-ended question are displayed in Table 4 for trainees and Table 5 for supervisors. For both groups the answers could be grouped into suggestions to improve test content and suggestions to improve educational impact.

**Table 4:** Results open question: how to improve postgraduate progress testing—trainees

*Test content*
Less factual/percentage questions (46)
Different question format (23)
More daily practice orientated questions (20)
Test questions should be of irrefutable content (13)
Free internet access (‘open book’) (12)
Clear description of study material 10)
*Educational impact*
Add literature references (8)
Release answers (5)
Unsatisfactory test scores should have consequences (4)

**Table 5:** Results of open question: how to improve test graduate progress testing—educational supervisors

*Test content*
Add open questions (3)
Yearly evaluation progress test by trainees (2)
Free internet access (‘open book’)(2)
Add questions that assess profound understanding (1)
Clear description of study material (1)
Test questions should be relevant in daily clinical practice (1)
Improve the array of questions: from simple to complex (1)
*Educational impact*
Test results should have consequences (4)
Organize test result evaluation sessions with educational supervisors (3)
Test should be obligatory to consultant obstetricians and gynaecologists as a part of re-certification (2)

Regarding test content: a plea for more practice-based, clinically relevant questions was frequently heard:

More relevant questions from daily practice, less percentages that are easily found on the internet when really needed (t)

Many trainees referred to the fact that current easily accessible resources on the internet have changed daily clinical practice, resulting in a lesser need for functional knowledge and factual recall. To them the test would become more valid by adding the possibility to access the internet during the test.

This sort of knowledge testing has become redundant, when in daily practice all information can be searched on the internet with two mouse clicks (t)

Trainees and educational supervisors suggested that educational impact could be improved by timely release of correct answers and literature references.

Display correct answers and literature references directly following the test (t)

Last, in both groups several participants stated that summative assessment with both clear standards and consequences for not passing the test would enhance the educational impact of the test.

Unsatisfactory test results should have consequences for the progress of a trainee (es)

National licensing exams with clear guidance on study material and consequences for failing the exams would be much better (t)

## Discussion

Postgraduate knowledge progress testing that is primarily intended to provide feedback and that can direct and steer learning is generally well accepted by both the trainees and educational supervisors in our study. However, the self-reported educational impact is limited; even though both trainees and supervisors indicate that test results should be part of the in-training assessment and personal learning plans, this is not put into practice. Furthermore, trainees query the relevance of test content. At the same time, both educational supervisors and trainees agree that mandatory progress testing should continue. In general we find that educational supervisors hold stronger views about the need for mandatory postgraduate progress testing, the importance to discuss test results during in-training assessments and the consequences of substandard performance.

### Acceptability

Both educational supervisors and postgraduate trainees indicate they accept assessment as a part of postgraduate training and endorse the interpretation of test scores. However, trainees do not consider the current progress test an important evaluation of their progress and most certainly do not want the test to have more serious consequences. This is in line with other studies that show that medical students prefer formative assessment to summative assessment [14, 15] as formative assessment is perceived to invoke less test-anxiety and encourage deeper learning.

### Limited educational impact

Even though our progress test was specifically introduced for its feedback properties under the premonition that test results would guide trainee learning, the self-reported educational impact, with the exception of first-year trainees, does not meet expectations. We think that there are several possible explanations for this finding.

### Quality of feedback

First of all, assessment can only be formative when used to improve performance. This implies that the information that is fed back needs to be sufficiently detailed and contain information on how performance can be improved and that trainees should be provided with an opportunity to show what they have learned [16, 17]. In our case both trainees and educational supervisors merely receive global feedback in the form of a grade on five different sub-domains, without information on which questions they answered incorrectly, where correct information can be found or how they can improve their performance. Furthermore, as the test is held only once a year and consists of a completely new set of questions every year, trainees do not get an opportunity to demonstrate what they have learned [18]. Additionally, there is sufficient evidence that feedback will only lead to performance improvement when recipients accept the feedback as valid [19, 20]. However our trainees question the representativeness and the practical applicability of the questions used in the test.

### Effect of grading

It has been noticed before that formative assessment is often not taken seriously as it is not perceived as high stakes; ‘it does not count’ [21]. Additionally, it has been shown that students find it hard to just receive feedback, with no indication of how they performed compared with preset standards or to their peers [22, 23]. However, it is also known that grading, even in the presence of extensive comments, hampers formative purposes [24, 25]. Our knowledge test is intended to be formative, but has a summative appearance: test scores are expressed numerically and a pass/fail judgement is given. It is possible that the summative appearance of the test leads trainees to ignore the feedback once they ‘pass’ the test. This finding may even be reinforced by the fact that test results are of no consequence to the trainees.

We can only guess why first-year trainees differed in their answers from older colleagues: perhaps they are still more aligned with the traditional, summative exams from most undergraduate curricula and consequentially perceive the test as a high-stakes judgment of their performance.

### Educational supervisors more strict?

The educational supervisors in this study appear stricter and tend to take progress test results more seriously. This may be explained by a more traditional background where assessment is concerned [14]. However, it can also be a reflection of the finding that educational supervisors who are involved in in-training assessments experience a need for objective criteria on which to base progress judgments [26, 27]. Our findings raise the question whether trainees are aware of the different stance of their educational supervisors regarding progress test results and whether this affects the trainee–educational supervisor relationship. We think more research is warranted into the background of in-between group differences regarding progress testing in postgraduate education.

### Putting the results into perspective

When only the results of the above-presented questionnaire are taken into consideration, one could get the impression that the utility of postgraduate knowledge progress testing will gain a lot from efforts to improve test quality. However, our progress test is surrounded by an extensive quality cycle, and previous efforts to improve test quality did not result in a better construct validity. Additionally we find it hard to accept that final-year trainees are content with knowing the correct answer to 60 % of the questions and filling out 33 % of test questions incorrectly. Hence we wonder whether it is possible that the perception of insufficient test quality is also used as a justification for not having to take test results too seriously.

Other studies show a measurable knowledge growth, or construct validity, when knowledge progress testing has a summative purpose or is linked firmly to a summative test [3, 28]. For this reason we recommend that efforts to improve the utility of formatively intended knowledge progress testing should have several aspects:

1. Improve test quality. This can be accomplished by re-using test questions that have shown a good discriminative power or involving more senior trainees and/or practising gynaecologists in the rating of item relevance.
2. Change the perception of the test from low to high stakes. This can be achieved by firmly embedding the test in the training programme. For example by incorporating strengths–weaknesses analysis and a personal learning plan based on test results as a mandatory element of the in-training assessment, by triangulating test results with other (performance-based) assessment and/or by organizing consultant-led discussion sessions during which all progress test questions are covered [29–31]. Implementing the last item will at the same time take care of the problem of deficient validity, as the relevance of the question becomes part of the discussion.
3. Provide an occasion to demonstrate improvement. It should be considered to either provide the opportunity to repeat the progress test or to link the formatively intended knowledge progress test to summative knowledge assessment in the form of licensing exams in Dutch postgraduate training.

## Strengths and limitations

The strength of this study is that we gained insight into the acceptability and educational impact of formatively intended postgraduate progress testing in medical education. Both trainees and supervisors were questioned and the high response rate made good triangulation possible.

Our results are somewhat limited by the fact that we used a non-validated questionnaire; however, as we reached the entire population of postgraduate trainees in O&G and their educational supervisors, we believe our results are sufficiently meaningful. Furthermore, we did not investigate whether (expected) poor test scores influenced trainees’ perceptions of the test. We also did not ask educational supervisors their opinion of the acceptability of the test content as we assumed not all would have sufficient recent information to judge test content. It would be interesting to find out, though, whether detailed knowledge of test content would generate different answers and how educational supervisors would rate test content.

On the whole it can be argued that we only managed to measure stakeholder satisfaction with progress testing, or Kirkpatrick level 1 [32]. Whereas, preferably, the impact of an educational intervention in postgraduate medical training should be measured at Kirkpatrick level 3 or 4: improvement of clinical performance or improved healthcare outcomes. However, it is notoriously difficult to measure the results of educational interventions at this level [12]. Besides, as Dutch postgraduate O&G training does not incorporate summative knowledge assessment, it is not possible to measure even Kirkpatrick level 2: improved learning. For this reason we were limited to measuring stakeholder satisfaction and self-reported impact on learning rather than measuring educational impact at the level where it counts: improved clinical performance and better patient care.

## Conclusion

General acceptability of formatively used postgraduate progress testing is good; however, educational impact is limited. We discuss the possible influence of feedback quality and grading on our findings. Furthermore we argue that for formative assessment to exert a genuine effect on learning, the assessment should have consequences for the trainee: either by firmly embedding the assessment in the training programme and/or by connecting formative assessment to a summative standard.

## Future research

In the light of the vast increase in formative assessment instruments in postgraduate medical training, it is of paramount importance to develop a validated instrument that can reliably measure acceptability and/or educational impact of formatively intended assessment.

Furthermore, the in-between group differences that we found for trainees and educational supervisors and the lack of educational impact warrant further, qualitative and in-depth research into the backgrounds of our findings.

## Essentials

- General acceptability of formatively used progress testing in postgraduate medicine is good.
- Educational impact of formatively used progress testing in postgraduate medicine in its current form is limited.
- To improve the educational impact of formatively intended progress testing the test (results) should have consequences, either by firmly embedding the test in the training programme or by linking the test to a high stakes, summative exam.
